# Biological activity of cinnamaldehyde, citronellal, geraniol and anacardic acid on *Haemonchus contortus* isolates susceptible and resistant to synthetic anthelmintics

**DOI:** 10.1590/S1984-29612023027

**Published:** 2023-06-19

**Authors:** Gracielle Araújo Frota, Valderlândia Oliveira dos Santos, Janaelia Ferreira Vasconcelos Rodrigues, Breno Reinaldo Oliveira, Laísa Bastos Albuquerque, Fernando Raul Correia de Vasconcelos, Adelino Carneiro Silva, Marcel Teixeira, Edy Souza de Brito, Jéssica Maria Leite dos Santos, Luiz da Silva Vieira, Jomar Patricio Monteiro

**Affiliations:** 1 Programa de Pós-graduação em Zootecnia, Universidade Estadual Vale do Acaraú – UVA, Sobral, CE, Brasil; 2 Programa de Pós-graduação em Microbiologia, Parasitologia e Patologia, Universidade Federal do Paraná – UFPR, Curitiba, PR, Brasil; 3 Centro Universitário Inta – UNINTA, Sobral, CE, Brasil; 4 Embrapa Caprinos e Ovinos, Sobral, CE, Brasil; 5 Embrapa Alimentos e Territórios, Maceió, AL, Brasil

**Keywords:** Cinnamaldehyde, citronellal, geraniol, anacardic acid, Haemonchus contortus, anthelmintic resistance, Cinamaldeído, citronelal, geraniol, ácido anacárdico, Haemonchus contortus, resistência anti-helmíntica

## Abstract

Parasitism by gastrointestinal nematodes is a challenge for small ruminant farming worldwide. It causes productive and economic losses, especially due to parasite resistance to conventional anthelmintics. Natural compounds with antiparasitic activity are a potential alternative for controlling these parasites especially when considering the widespread occurrence of anthelmintic resistance. Our objective was to evaluate the activity of anacardic acid, geraniol, cinnamaldehyde and citronellal on *Haemonchus contortus* isolates with different levels of anthelmintic resistance profiles. These compounds were tested using egg hatch assays (EHAs), larval development tests (LDTs) as well as LDTs on mini-fecal cultures, on the *Haemonchus contortus* isolates Kokstad (KOK-resistant to all anthelmintics), Inbred-Strain-Edinburgh (ISE-susceptible to all anthelmintics) and Echevarria (ECH-susceptible to all anthelmintics). Effective concentrations to inhibit 50% (EC_50_) and 95% (EC_95_) of egg hatching and larval development were calculated. Results for EHA and LDT for all tested compounds, considering EC_50_ and EC_95_ values, showed low variation among the studied isolates with most RF values below 2x. All studied compounds showed efficacy against egg hatching and larval development of *H. contortus* isolates regardless of anthelmintic resistance profiles. The compounds with the smallest EC_50_ and EC_95_ values were cinnamaldehyde and anacardic acid making them promising candidates for future *in vivo* studies.

## Resumo

A infecção por nematoides gastrintestinais é um dos principais desafios na produção de pequenos ruminantes e ocasiona perdas produtivas, principalmente, devido à resistência anti-helmíntica. Bioativos com atividade anti-helmíntica são potencial alternativa para o controle desses parasitos em especial, considerando-se a ampla incidência de resistência anti-helmíntica nos rebanhos. O objetivo deste estudo foi avaliar a atividade biológica do ácido anacárdico, geraniol, cinamaldeído e citronelal em isolados de *Haemonchus contortus* com diferentes perfis de resistência anti-helmíntica. Foram realizados testes de eclosão de ovos (TEO), testes de desenvolvimento larvar (TDL) e TDLs em minicoproculturas, utilizando-se o isolado Kokstad (resistente a todos os anti-helmínticos), o isolado Inbred-Strain-Edinburgh (suscetível) e o isolado Echevarria (suscetível). Foram calculadas as concentrações efetivas para inibir 50% (CE_50_) e 95% (CE_95_) da eclodibilidade dos ovos e do desenvolvimento larvar. Resultados de TEO e TDL apresentaram baixa variação entre os diferentes isolados para um mesmo composto testado com fatores de resistência geralmente abaixo de 2x. Todos os compostos estudados mostraram eficácia contra a eclosão de ovos e desenvolvimento larvar de isolados de *H. contortus* independente do perfil de resistência anti-helmíntica dos mesmos. Os compostos que apresentaram atividade nas menores concentrações foram cinamaldeído e ácido anacárdico, sendo estes os componentes mais promissores para futuros estudos *in vivo*.

## Introduction

Gastrointestinal nematodes are the main cause of productive and economic losses in small ruminant production systems. *Haemonchus contortus* is considered to be the most pathogenic parasite and can cause fatal anemia in animals with a high parasite load (Zajac & Garza, 2020; Salgado & Santos, 2016).

The main way of controlling these nematodes is through antiparasitic drugs. However, use of these substances has become compromised since many populations of parasites have already developed anthelmintic resistance (Gaudin et al., 2016). However, resistance mechanisms differ at the genetic level for different classes of anthelmintic compounds (Bartram et al., 2012). Considering this scenario, the prospection of new compounds with anthelmintic activity, that may bypass commercial anthelmintic resistance mechanisms, may be a viable alternative in scenarios with decreased efficacy of commercial compounds (Molento et al., 2020). Considering this scenario, the prospection of new compounds with anthelmintic activity may be a viable alternative to the commercial compounds with decreased efficacy (Molento et al., 2020). In addition, the demand for animal products from sustainable organic systems has increased (Zajac & Garza, 2020), thus making it necessary to search for new control alternatives (Barone et al., 2018).

Essential oils from aromatic plants have been studied for their therapeutic and nematocidal effects. Cinnamon (*Cinnamomum* spp.) belongs to the Lauraceae family, from South and Southeast Asia, and is traditionally used in cooking and as a medicinal plant (Bakar et al., 2020). Its major component, cinnamaldehyde, has been shown to be effective against some species of fungi (Doyle & Stephens, 2019) and has antimicrobial activity on bacteria of the quinolone-resistant Enterobacteriaceae family (Dhara & Tripathi, 2020), on eggs of *H. contortus* (Katiki et al., 2017) and on nematodes such as *Caenorhabditis elegans* (Lu et al., 2020).

Citronellal is the major component of the essential oil of *Eucalyptus citriodora*. It has proven effectiveness as an antimicrobial (Bezerra et al., 2019), repellent action against *Anopheles gambiae* s.s. (Wu et al., 2020), action against *Staphylococcus aureus* and *Escherichia coli* biofilms (Borges et al., 2017) and action against nematode eggs and larvae (Araújo-Filho et al., 2018).

Geraniol is present in several plants such as *Cymbopogon martini*, *Pelargonium graveolens*, *Rosa damascena*, *Rosa centifolia*, *Cymbopogon nardus* and *Cymbopogon winterianus* (Chen & Viljoen, 2010; Lira et al., 2020). It has been shown to have insecticidal, antioxidant, anti-inflammatory, antifungal, antibacterial and antitumour activities (Singh et al., 2016; Wang et al., 2016; Tabari et al., 2017; Qi et al., 2018; Lin et al., 2021).

Cashew production is significant in Brazil and anacardic acid is the major component of the cashew nutshell liquid. It has been the focus of several studies in the past decades with biological properties such as antitumor, antibacterial, molluscidal and anthelmintic (Sullivan et al., 1982; Muroi & Kubo, 1996; Hemshekhar et al., 2012; Tan et al., 2017; Dube et al., 2021).

The aim of this study was to evaluate the biological activity of cinnamaldehyde, citronellal, geraniol and anacardic acid against *H. contortus* isolates susceptible and resistant to synthetic anthelmintics.

## Material and Methods

### *Haemonchus contortus* isolates

*Haemonchus contortus* isolates with differing resistance and susceptibility to anthelmintics were used in this study. The Inbred-Strain-Edinburgh isolate (ISE) was used as a susceptibility reference in relation to all commercial anthelmintics (Roos et al., 2004); the Kokstad isolate (KOK) was used as a multidrug resistance reference (Barrère et al., 2014); and the native isolate Echevarria (ECH), with no history of resistance, from a farm in Rio Grande do Sul, Brazil (Echevarria et al., 1991), was compared against the other two isolates.

Twelve adult sheep were used after deworming (ivermectin, 200 μg/kg; oxfendazole, 5 mg/kg; monepantel, 2.5 mg/kg; and levamisole, 7.5 mg/kg), which was confirmed through fecal egg counts (sensitivity of 25 eggs per gram) and fecal cultures showing no eggs or larvae. The animals were divided into four groups (n = 3) and each group was infected with a single *H. contortus* isolate, while the fourth group was kept as an infection-free control. Each animal received 5,000 third stage (L3) infective larvae orally. The experimental infections were monitored through fecal egg counts every week and fecal cultures every two weeks. The animals from each group were confined in isolated pens with slatted floors and given corn and sorghum silage and ration following NRC’s requirements (NRC, 2007) as well as water *ad libitum*.

### Organic compounds

Pure cinnamaldehyde, citronellal and geraniol used in all tests were purchased from Sigma-Aldrich (St. Louis, MO, USA). Anacardic acid from the industrial processing of cashew nuts was purified at Embrapa Agroindústria Tropical (Fortaleza, CE, Brazil). Chemical analyzes were carried out at the CNPAT Laboratory of Natural Product Chemistry in Fortaleza-CE. Qualitative tests were performed as previously described (Matos, 1997). Quantitative tests were performed by chromatography on an HPLC apparatus using a mixture of ethyl acetate and toluene 10:90 V/V as eluent and gas chromatography with helium gas in the mobile phase. The isolated substances were identified by 13C and 1H Nuclear Magnetic Resonance (NMR) and Infrared. The identification of essential oils was performed using gas chromatography coupled to mass spectrometry, while the quantification was performed using gas chromatography with a flame ionization detector (DIC), both using nitrogen as carrier gas.

### Egg hatch assay (EHA)

Egg recoveries and EHAs were performed as per the recommendations from the World Association for the Advancement of Veterinary Parasitology (WAAVP) (Coles et al., 1992). For EHAs, 250 μl of egg suspension (~100 eggs/100 μl) was used, and 250 μl of treatment solution (cinnamaldehyde or citronellal at the desired concentration) was added, to make up a final volume of 500 μl/well. The plates were incubated at 27 ± 1 °C for 48 h and at least 100 eggs and L1 larvae were counted in each well using an inverted microscope.

Cinnamaldehyde (final concentrations: 378.33, 189.16, 94.58, 47.29, 23.65 and 11.82 μM), citronellal (final concentrations: 25,931.93, 6,482.98, 1,620.75, 405.19, 101.30, 25.32, 6.33 and 1.58 μM) and geraniol (final concentrations: 6,480, 4,860, 3,240, 1,620, 1,210, 810, 410 and 200 µM) were diluted in 0.25% Tween 80. Anacardic acid (final concentrations: 143.46, 71.73, 35.86, 17.93 and 8.96 µM) was diluted in 0.3% DMSO. Cinnamaldehyde and citronellal tests were done with six replicates for each concentration. Anacardic acid and geraniol tests were done with five replicates. The negative controls consisted of 0.25% Tween 80 for all compounds except anacardic acid which was 0.3% DMSO and the positive controls contained thiabendazole (TBZ) (0.025 mg/mL, diluted in 0.3% DMSO).

### Larval development test (LDT)

All the LDTs were performed in mini-fecal cultures as previously described (Camurça-Vasconcelos et al., 2007), with minor modifications except for anacardic acid which was evaluated by the regular LDT (Coles et al., 2006). For the LDT in mini-fecal cultures, the eggs recovered were incubated for 24 hours at 27 ± 1 °C to obtain L1 larvae. Afterwards, 500 μL of the L1 larvae solution (~ 250 L1 larvae), plus 100 μL of nutrient medium (lyophilized *Escherichia coli*, yeast extract and amphotericin B 0.49 µg/mL) and 600 μL of the treatment (cinnamaldehyde, citronellal or geraniol) were added to two grams of feces (from parasite-free animals). This mixture was homogenized and incubated for another 6 days at 27 ± 1 °C and ideal humidity (> 80%). L3 larvae recoveries were done as previously described (Roberts & O'Sullivan, 1950). The L3 larvae thus recovered were transferred to 24-well plates and counted using an inverted microscope.

Cinnamaldehyde (final concentrations: 22,699.75, 15,133.17, 11,349.87, 7,566.68, 5,674.93, 3,783.29 and 1,891.64 μM) was diluted in 0.4% Tween 80, citronellal (final concentrations: 64,829.82, 32,414.91, 16,207.45, 8,103.72, 4,051.86, 2,025.93, 1,012.96 and 506.48 μM) was diluted in 1.1% Tween 80 and geraniol (final concentrations: 38,897.89, 32,414.91, 25,931.92, 19,448.94, 16,207.45, 9,724.47 and 6,482.98 µM) was diluted in 0.5% Tween 80. Anacardic acid (final concentrations: 22.95, 11.48, 5.74, 4.30, 2.87, 2.15 and 1.43 µM) was diluted in 0.3% DMSO. All the tests were performed with five replicates for each concentration. The test with anacardic acid had six replicates for each treatment. The controls comprised of a test with water and nutrient medium only; an untreated control (larvae, nutrient medium and water); a negative control (larvae, nutrient medium and Tween 80 or DMSO at above mentioned concentrations); and a positive control using ivermectin at 8 µg/mL.

### Data analysis and statistical analysis

Treatment efficacies per well in EHAs were calculated using the following formula:


(egg counts/egg counts+L1 larvae counts) x 100 (1)

Treatment efficacies in LDTs were calculated using the following formula (Araújo-Filho et al., 2019):


$$\left({\bar{x}}_{negative\;ctrl.\;\;L3\;larvae}-L3\;larvae\;in\;treatment/{\bar{x}}_{negative\;ctrl.\;\;L3\;larvae}\right)\;x\;100$$ (2)

The effective concentrations for inhibiting 50% (EC_50_) and 95% (EC_95_) of egg hatching and larval development were calculated using nonlinear regression analysis (Bates & Watts, 1988). Resistance factors for EC_50_ and EC_95_ represent the ratios between KOK or ECH isolates in relation to the ISE isolate (RF_50_= EC_50_ for isolates X/EC_50_ for the ISE isolate; RF_95_= EC_95_ for isolates X/EC_95_ for the ISE isolate). Effective concentrations generated by the EHAs and LDTs were compared by Two-way ANOVA and Tukey’s test for multiple comparisons. Within the EHA and LDT data sets, the comparisons were done for each tested compound and *H. contortus* isolate and differences were considered significant at *p* < 0.05. All statistical analysis were done using Prism 6 (v 6.07, 2015, GraphPad Software, Inc., Boston, MA, USA) (GraphPad Software, 2015).

## Results

**Figure 1:**
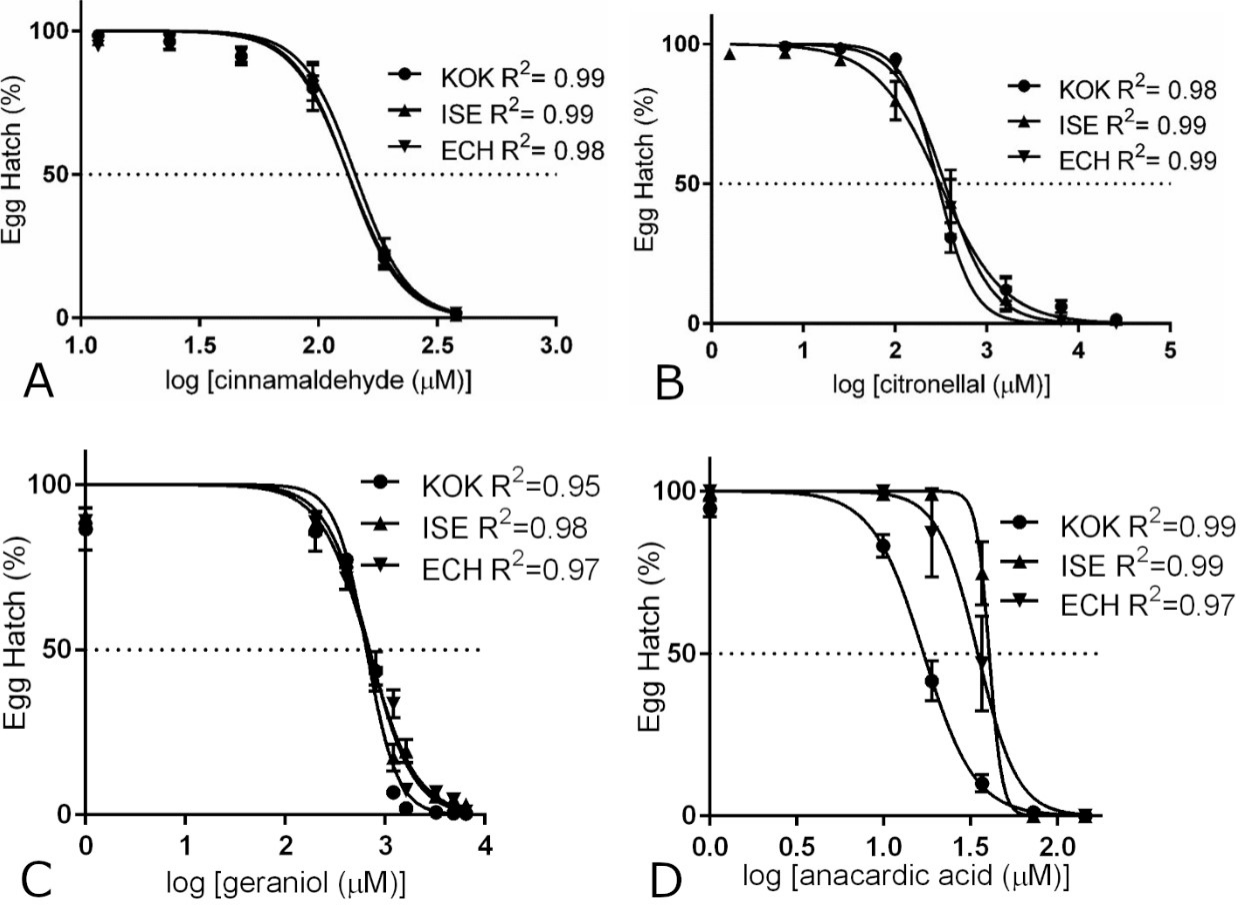
Nonlinear regression curves of EHAs with cinnamaldehyde (panel A), citronellal (panel B), geraniol (panel C) and anacardic acid (panel D) for the *H. contortus* isolates KOK, ISE and ECH. R^2^: coefficient of determination.

**Figure 2:**
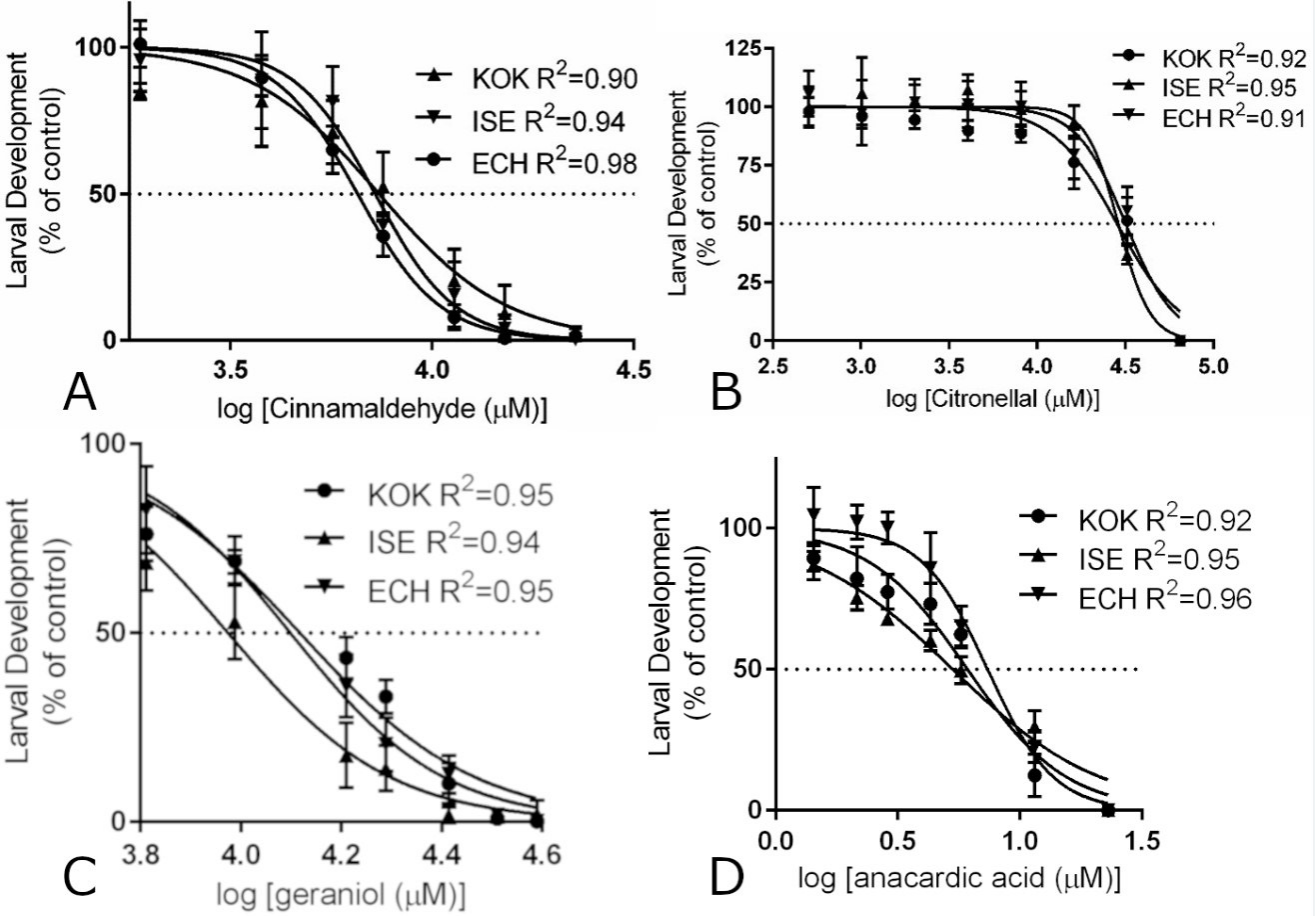
Nonlinear regression curves of LDTs with cinnamaldehyde (panel A), citronellal (panel B), geraniol (panel C) and anacardic acid (panel D) for the *H. contortus* isolates KOK, ISE and ECH. R^2^: coefficient of determination.

The results from EHAs and LDTs for all tested compounds on the *H. contortus* isolates KOK, ISE and ECH are shown in Figures 1 and 2. All non-linear regression analysis resulted in coefficients of determination (R^2^) above 0.90 showing a good fit of the data against the calculated regression curves (Figures 1 and 2). Both EC_50_ and EC_95_ for EHAs and LDTs were very similar between isolates for any given studied compound and were never over or below 3 times the values for the ISE isolate. Therefore, the comparison between the different isolates for each compound did not show statistically significant differences, on the other hand, the comparison between the different compounds for each isolate showed significant differences in terms of concentration (p < 0.05) with the exception of citronellal and geraniol EC_95_ for the ISE isolate which was not significant.

**Figure 3:**
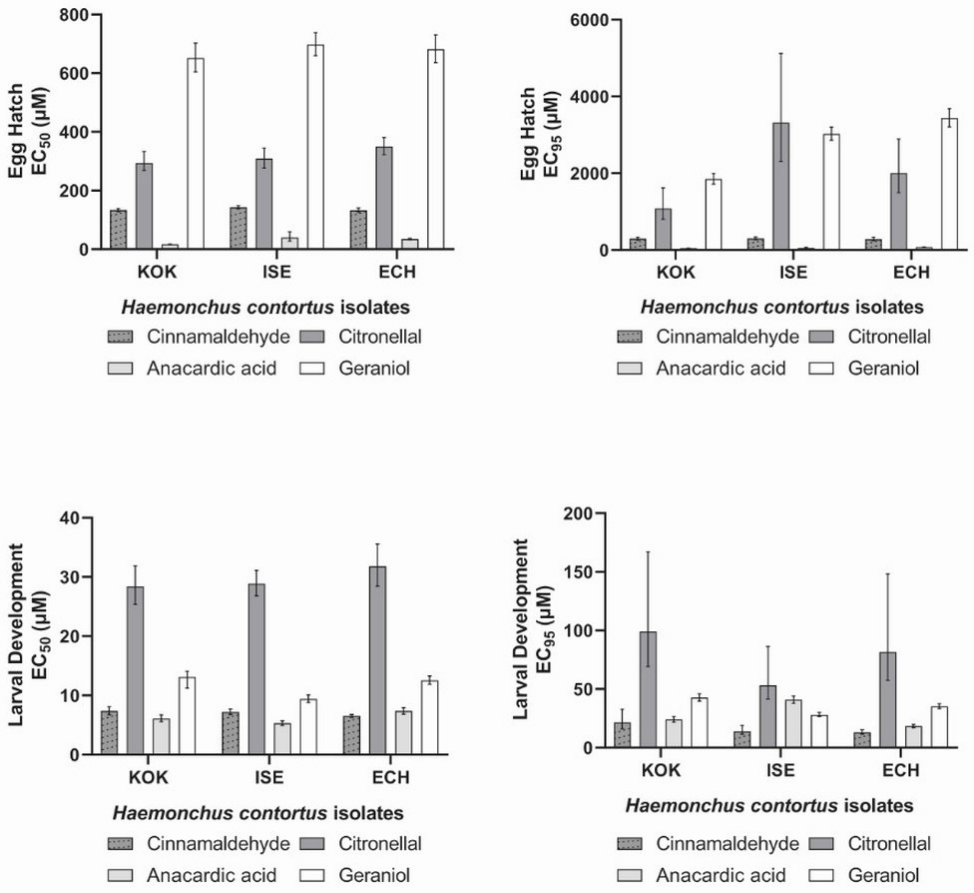
Effective concentrations at 50% and 95% in egg hatching and larval development tests with cinnamaldehyde, citronellal, geraniol and anacardic acid for *H. contortus* KOK, ISE and ECH isolates. The bars show 95% confidence intervals.

Figure 3 shows the effective concentrations of inhibition of egg hatching and larval development among the different compounds against the three isolates used in this work KOK, ISE and ECH. It can be observed that the compounds requiring the smallest amounts for egg hatching and larval development inhibition were cinnamaldehyde and anacardic acid (*p* < 0.05).

### Egg hatch assay (EHA)

Anacardic acid presented the lowest EC_50_ (16.97 µM) and EC_95_ (46.12 µM) values when tested against the resistant isolate KOK. In general, the lowest EC_50_ and EC_95_ are observed when tested against this same isolate, except for cinnamaldehyde, where the lowest concentrations were observed in the test with the ECH isolate (*p* < 0.05). Geraniol was the compound that required the largest amounts in terms of egg hatching inhibition with values of 697.90 µM (EC_50_) and 3,440.26 µM (EC_95_) for the ISE and ECH isolates, respectively (Table 1).

**Table 1:** Effective concentrations of cinnamaldehyde, citronellal, anacardic acid and geraniol for inhibiting 50% and 95% of egg hatching (EC_50_ and EC_95_), confidence intervals (95% CI) and resistance factors for *Haemonchus contortus* isolates KOK, ISE and ECH.

	*H. contortus* isolates	EC_50_ (μM)	95% CI	RF_50_	EC_95_ (μM)	95% CI	RF_95_
	KOK	133.6	(128.5 - 138.9)	0.94	295.6	(263.5 - 337.6)	0.98
Cinnamaldehyde	ISE	142.8	(137.7 - 148.1)	1.00	301.1	(270.4 - 340.8)	1.00
	ECH	133.2	(126.7 - 140.0)	0.93	283.2	(245.8 - 335.8)	0.94
	KOK	294.5	(268.6 - 322.9)	0.95	1,083.1	(801.9 - 1,615.2)	0.33
Citronellal	ISE	308.7	(276.6 - 344.5)	1.00	3,317.3	(2,300.2 - 5,120.1)	1.00
	ECH	350.6	(322.4 - 381.3)	1.14	2,008.2	(1,494.2- 2,892.9)	0.61
	KOK	16.97	(16.30 - 17.67)	0.42	46.12	(44.30 - 48.02)	0.89
Anacardic acid	ISE	40.44	(27.67 - 59.12)	1.00	52.02	(35.60 - 76.10)	1.00
	ECH	34.79	(32.51 - 37.23)	0.86	75.23	(70.29 - 80.50)	1.45
	KOK	651.60	(604.3 - 702.6)	0.93	1,851.14	(1,716.77 - 1,996.03)	0.61
Geraniol	ISE	697.90	(659.9 - 738.1)	1.00	3,028.71	(2,863.80 - 3,203.17)	1.00
	ECH	681.70	(635,9 - 730,8)	0.98	3,440.26	(3,209.13 - 3,688.05)	1.14

EC_50_: effective concentration for inhibiting 50% of egg hatching; EC_95_: effective concentration for inhibiting 95% of egg hatching; 95% CI: 95% confidence interval; RF_50_: resistance factor for EC_50_ (EC_50_ of isolate X/EC_50_ for the ISE isolate); RF_95_: resistance factor for EC_95_ (EC_95_ of isolate X/EC_95_ for the ISE isolate).

### Larval development tests (LDTs)

Larval development was the least affected by the tested compounds requiring concentrations in the millimolar range except for anacardic acid with EC_50_ and EC_95_ values always below 50 μM (*p* < 0.05). As in the EHA, anacardic acid was the compound that required the smallest amounts for larval development inhibition. The lowest EC_50_ (5.31 µM) and EC_95_ (18.34 µM) values were observed in the ISE and ECH isolates, respectively. In contrast, citronellal was the compound that showed the highest EC_50_ and EC_95_ values among all compounds and in all studied isolates (Figure 3) with EC_50_ values above 28,000 µM and EC_95_ values above 53,000 µM (Table 2). It is noteworthy that the anthelmintic profiles of the studied isolates had no influence in both tests observed results as the EC_50_ and EC_95_ values obtained were very similar across isolates and the resistance factor values, based on the ISE isolate, calculated was rarely above 2 (Tables 1 and 2).

**Table 2:** Effective concentrations of cinnamaldehyde, citronellal, anacardic acid and geraniol for inhibiting 50% and 95% of larval development (EC_50_ and EC_95_), confidence intervals (95% CI) and resistance factors for *Haemonchus contortus* isolates KOK, ISE and ECH.

	*H. contortus* isolates	EC_50_ (μM)	95% CI	RF_50_	EC_95_ (μM)	95% CI	RF_95_
	KOK	7,396	(6,766 - 8,083)	1.02	21,594	(16,127 - 32,738)	1.53
Cinnamaldehyde	ISE	7,232	(6,787 - 7,706)	1.00	14,115	(11,510 - 19,164)	1.00
	ECH	6,542	(6,319 - 6,772)	0.91	13,031	(11,529 - 15,183)	0.92
	KOK	28,422	(25,363 - 31,850)	0.98	99,284	(69,237 - 167,158)	1.86
Citronellal	ISE	28,883	(26,809 - 31,117)	1.00	53,319	(41,555 - 86,254)	1.00
	ECH	31,816	(28,463 - 35,565)	1.10	81,654	(57,513 - 148,312)	1.53
	KOK	6.11	(5.588 - 6.710)	1.15	24.01	(21.85 - 26.38)	0.59
Anacardic acid	ISE	5.31	(4.929 - 5.719)	1.00	40.85	(37.93 - 44.01)	1.00
	ECH	7.38	(6.858 - 7.931)	1.39	18.34	(17.06 - 19.72)	0.45
	KOK	13,119	(11,210 - 14,096)	1.39	42,670	(39,741 - 45,880)	1.52
Geraniol	ISE	9,430	(8,822 - 10,081)	1.00	28,154	(26,338 - 30,097)	1.00
	ECH	12,560	(11,867 - 13,293)	1.33	35,318	(33,369 - 37,379)	1.26

EC_50_: effective concentration for inhibiting 50% of larval development; EC_95_: effective concentration for inhibiting 95% of larval development; 95% CI: 95% confidence interval; RF_50_: resistance factor for EC_50_ (EC_50_ of isolate X/EC_50_ for the ISE isolate); RF_95_: resistance factor for EC_95_ (EC_95_ of isolate X/EC_95_ for the ISE isolate).

## Discussion

The lethal dose of cinnamaldehyde for the free-living nematode *C. elegans* was determined as 6,058.27 μM for 4 hours, which induced multiple gene expression changes that were mainly involved in glutathione metabolism (Lu et al., 2020). *Ascaris suum* exposed to *Cinnamomum verum* extract (7.8% cinnamaldehyde) showed damage to the muscle layer and digestive tract, without many alterations to the parasite cuticle (Williams et al., 2015). This suggests that the mechanism of action is through changes to internal structures after ingestion. In addition to the effects on small-ruminant and pig nematodes, cinnamaldehyde was also effective on the soybean nematode *Meloidogyne incognita* (Jardim et al., 2018). The essential oil of *Cinnamomum cassia* (83% cinnamaldehyde) caused 100% mortality and immobility of the larvae at a concentration of 389.37 μM. During evaluations on supplying cinnamaldehyde in the diet of dairy cows, this compound proved to be safe at concentrations of 0.2 to 4 mg/kg of body weight. It did not cause changes to feed consumption, ruminal fermentation, ruminal pH, milk composition or milk production and did not affect the digestion of nutrients in the diet (Chapman et al., 2019). This suggests that this compound is also safe for carrying out *in vivo* tests with small ruminants. The effect of cinnamaldehyde on egg hatching was previously studied in a multidrug-resistant *H. contortus* isolate (resistant to ivermectin, moxidectin, closantel, albendazole, levamisole phosphate and trichlorfon) resulting in an EC_50_ of 136.19 μM (Katiki et al., 2017). We observed similar EC_50_ and EC_95_ values for the isolates studied here (Figures 1 and 3; Table 1). In the same manner, the LDT results were also equivalent for all the isolates studied (Figures 2 and 3; Table 2) but required concentrations to impair larval development were in general 50 times higher than the values obtained in the EHA. These results are more in line with the values obtained against adult *C. elegans* as above mentioned. Since the results were similar for nematodes with different profiles of anthelmintic resistance, it may be that the commercial anthelmintic resistance mechanisms do not affect the action of cinnamaldehyde on *H. contortus* isolates. As cinnamaldehyde has shown effects on different species of nematodes inhabiting different environments and has the potential to be safe for small ruminants, it remains as an interesting candidate for further studies.

Citronellal and geraniol were the compounds requiring much higher concentrations in the hundreds or thousands micromolar to inhibit egg hatching and larval development. The effects of citronellal on egg hatching and larval development were also equivalent for the isolates studied (Figure 1; Tables 1 and 2). The essential oil of *Eucalyptus citriodora*, containing 71.77% beta-citronellal was also effective against *H. contortus* egg hatching and larval development with EC_50_ values of 5.3 and 12.61 mM of citronellal content for EHA and LDT respectively (Macedo et al., 2011). In the same manner, citronellal was previously shown to have ovicidal activity with EC_50_ values of 1.95 mM for the ISE isolate and 2.59 for the KOK isolate. It also impaired larval development with EC_50_ values of 14.91 mM and 15.56 mM for the same *H. contortus* isolates respectively (Araújo-Filho et al., 2018). Our results (Figures 1 and 2 and Tables 1 and 2) are in general agreement with this data as we also found lower EC_50_ values for egg hatch inhibition and higher values for larval development impairment. Fecal egg count reduction tests using *E. citriodora* essential oil (67.5% citronellal) and nanoencapsulation reduced the egg counts by 40.5% and 55.9% for the free oil and nanoencapsulated oil respectively (Ribeiro et al., 2014). Another study showed fecal egg count reduction results of 69.5% for *E. citriodora* essential oil and no significant reduction with citronellal alone (Araújo-Filho et al., 2019). It appears that, despite having similar *in vitro* anthelmintic activity against *H. contortus* isolates with differing resistance profiles, citronellal alone did not show any efficacy *in vivo* against one field population. Thus, further *in vivo* studies should be carried out to check if this is the case for other field populations and *H. contortus* isolates as well.

Essential oils from *Cymbopogon schoenanthus* and *Cymbopogon martinii*, with 62.5% and 81.4% geraniol respectively, showed *in vitro* anthelmintic activity against a field population of gastrointestinal nematodes (95% *H. contortus* and 5% *Trichostrongylus* spp.). Egg hatching was inhibited at EC_50_ concentrations of 162.08 µM and 686.29 µM of geraniol for *C. schoenanthus* and *C. martinii* essential oils respectively. Larval development was impaired at EC_50_ concentrations of 243.11 µM and 791.57 µM of geraniol for *C. schoenanthus* and *C. martinii* essential oils respectively (Katiki et al., 2011). We obtained similar EHA results to the *C. martinii* essential oil which contains more geraniol than *C. schoenanthus* essential oil. On the other hand, our LDA EC_50_ resulted in higher amounts of geraniol than any of the *Cymbopogon* essential oils. Most probably, other components in these oils also possess anthelmintic activity especially in the case of larval development. Similar LC_50_ values for geraniol were obtained against *C. elegans* (432.42 µM) (Kumaran et al., 2003). Motility impairment by geraniol was also observed against *H. contortus*, *Trichostrongylus axei* and *Teladorsagia circumcincta* at 2% (*v/v*) with decreased effects against *Trichostrongylus colubriformis* and *Cooperia oncophora* (Helal et al., 2020). Resistance factors were very close to 1 in all cases, considering the different isolates studied here, suggesting that the commercial anthelmintic resistance mechanisms did not act on geraniol (Tables 1 and 2). Among the compounds tested in this study, geraniol required higher concentrations to attain an observable effect in EHAs and LDTs and, as far as we know, this is the first report of geraniol effects against multiple *H. contortus* isolates. Finally, *C. schoenanthus* essential oil had no significant *in vivo* effect in fecal egg counts from lambs when compared to controls (Katiki et al., 2012) and it remains to be tested whether geraniol alone will behave in the same manner.

In this study, anacardic acid was the compound that inhibited egg hatching and larval development at the lowest concentrations with EC_50_ and EC_95_ values always below 100 μM regardless of the isolate (Figure 3). Anacardic acid is extracted from the cashew nutshell liquid. There are few studies of its action against *H. contortus* using the extract from the nutshell and dry cashew apple fiber as a feed alternative for sheep. Cashew apple fiber containing 3.5% anacardic acid, among other compounds, was given as an alternative feed for 28 days to sheep that were experimentally infected with a multi-resistant *H. contortus* isolate. Egg counts throughout a period of 63 days showed a mean reduction of 60% in the group that was fed cashew apple fiber and anacardic acid was among the compounds postulated to have anthelmintic activity (Lopes et al., 2018). Hydroalcoholic extracts from the cashew nutshell were also tested for activity against egg hatching, larval motility, and adult motility in a natural population of gastrointestinal nematodes (99% *Haemonchus contortus*) with LD_50_ doses at 0.0258, 0.196 and 1.0365 mg/ml respectively (Davuluri et al., 2020). It is difficult to compare studies with purified compounds with studies that used extracts due to the presence of multiple substances in the extracts. Our results with anacardic acid match the results using cashew extracts but larval development was more sensitive than egg hatching with lower EC_50_ and EC_95_ doses. Discrepancies could be due to the effect of other compounds in the extracts. The above-mentioned studies also suggest that anacardic acid could have *in vivo* activity. Of all tested compounds, anacardic acid was the one with lower EC_50_ and EC_95_ values in our *in vitro* tests and RF values of the studied isolates were usually below 2x in comparison with the ISE isolate. This suggests that, like the other compounds studied here, anacardic acid is not affected by commercial anthelmintic resistance mechanisms. This added to the fact that Brazil is a cashew apple and cashew nut producer with over 45,000 metric tons of fruit and 51,000 metric tons of nuts produced in 2017 (IBGE, 2017a; IBGE, 2017b) places anacardic acid as a promising candidate for future *in vivo* tests.

## Conclusion

In conclusion we demonstrated the *in vitro* anthelmintic activities of several pure compounds against eggs and larvae of *H. contortus* isolates with differing anthelmintic resistance profiles. Anacardic acid and cinnamaldehyde appear to be promising candidates for future *in vivo* tests. In addition, resistance to commercial anthelmintics do not appear to affect the action of the tested compounds.
